# Supplementary material for: Decreased Associated Risk of Gout in Diabetes Patients with Uric Acid Urolithiasis
Source: J Clin Med. 2019 Sep 25;8(10):1536. doi: 10.3390/jcm8101536 (PMC6832126; doi:10.3390/jcm8101536)
Supplement: Supplementary file 1 [file jcm-08-01536-s001.pdf]

Journal of Clinical Medicine

Table S1. Difference in clinical features and laboratory findings between uric acid urolithiasis patients with and without gout (including metabolic syndrome and uric acid medications).

	Without gout (N=75)	With gout (N=24)	p value
	n (%)	n (%)	
**Age**			
≤ 60	27 (36.00)	12 (50.00)	0.326
> 60	48 (64.00)	12 (50.00)	
median (IQR)	65.00 (57.00, 72.00) (n=75)	60.50 (49.50, 73.00) (n=24)	0.363 [a]
**Gender**			
Female	12 (16.00)	0 (0.00)	0.036 [b]
Male	63 (84.00)	24 (100.00)	
**BMI (kg/m²)**			
mean ± SD	25.14 ± 4.52 (n=60)	26.70 ± 3.21 (n=22)	0.141
**Uric acid (mg/dl)**			
mean ± SD	7.14 ± 1.55 (n=38)	7.19 ± 1.71 (n=16)	0.905
**Urine pH**			
median (IQR)	6.00 (5.00, 6.00) (n=72)	6.00 (5.25, 6.00) (n=24)	0.541 [a]
**Creatinine (mg/dL)**			
≤ 1.5	56 (75.68)	13 (54.17)	0.080
> 1.5	18 (24.32)	1 (45.83)	
median (IQR)	1.18 (0.96, 1.46) (n=74)	1.38 (1.12, 1.84) (n=24)	0.052 [a]
**eGFR (mL/min/1.73m2)**			
< 45	14 (18.92)	10 (41.67)	0.048
≥ 45	60 (81.08)	14 (58.33)	
median (IQR)	65.67 (50.31, 77.07) (n=74)	55.56 (39.03, 70.37) (n=24)	0.167 [a]
**Cholesterol (mg/dL)**			
mean ± SD	176.27 ± 51.95 (n=30)	185.83 ± 37.38 (n=12)	0.566 [b]
**Triglyceride (mg/dL)**			
median (IQR)	101.00 (75.00, 122.00) (n=30)	157.00 (124.50, 176.50) (n=12)	0.021 [a]
**HbA1C (%)**			
median (IQR)	6.00 (5.70, 6.90) (n=34)	5.80 (5.40, 6.60) (n=15)	0.297 [a]
**Diabetes mellitus**			
No	49 (65.33)	23 (95.83)	0.003 [b]
Yes	26 (34.67)	1 (4.17)	
**Metabolic syndrome**			
No	67 (89.33)	22 (91.67)	1.000 [b]
Yes	8 (10.67)	2 (8.33)	
**Hypertension**			
No	41 (54.67)	11 (45.83)	0.603
Yes	34 (45.33)	13 (54.17)	
**Cardiovascular disease**			
No	61 (81.33)	22 (91.67)	0.344 [b]
Yes	14 (18.67)	2 (8.33)	
**Hyperlipidemia**			
No	18 (60.00)	5 (41.67)	0.462
Yes	12 (40.00)	7 (58.33)	
**Thiazide**			
No	71 (94.67)	24 (100.00)	0.570 [b]
Yes	4 (5.33)	0 (0.00)	
**Benzbromarone**			
No	74 (98.67)	16 (66.67)	<0.001 [b]
Yes	1 (1.33)	8 (33.33)	

[a] Mann-Whitney U test. [b] Fisher's exact test.

Table S2. Logistic regression analysis of risk factors for Gout among uric acid urolithiasis patients (including metabolic syndrome and uric acid medications).

	Crude OR (95 % CI)	p-value	Adjusted OR1 [a] (95 % CI)	p-value	Adjusted OR2 [a] (95 % CI)	p-value
**Age**	0.99 (0.95-1.02)	0.393				
**Age group**						
≤ 60	Ref.					
> 60	0.56 (0.22-1.42)	0.225				
**Gender**						
Female	Ref.					
Male	9.65 (0.49-189.89)	0.136 [a]				
**BMI (kg/m²)**	1.10 (0.97-1.24)	0.142				
**Uric acid (mg/dl)**	1.02 (0.70-1.49)	0.903				
**Urine pH**	1.33 (0.69-2.58)	0.392				
**Creatinine (mg/dL)**	1.48 (0.78-2.78)	0.231				
**Creatinine group**						
≤ 1.5	Ref.				Ref.	
> 1.5	2.63 (1.01-6.89)	0.049			3.00 (0.80-11.24)	0.102
**eGFR (mL/min/1.73m2)**	0.99 (0.97-1.01)	0.233				
**eGFR group**						
≥ 45	Ref.		Ref.			
< 45	3.06 (1.13-8.31)	0.028	3.92 (0.97-15.91)	0.056		
**Cholesterol (mg/dL)**	1.00 (0.99-1.02)	0.556				
**Triglyceride (mg/dL)**	1.01 (1.00-1.02)	0.231				
**HbA1C (%)**	1.07 (0.68-1.67)	0.774				
**Diabetes mellitus**						
No	Ref.		Ref.		Ref.	
Yes	0.08 (0.01-0.64)	0.017	0.12 (0.02-0.73)	0.022	0.12 (0.02-0.75)	0.023
**Metabolic syndrome**						
No	Ref.					
Yes	0.76 (0.15-3.86)	0.742				
**Hypertension**						
No	Ref.					
Yes	1.43 (0.57-3.59)	0.452				
**Cardiovascular disease**						
No	Ref.					
Yes	0.40 (0.08-1.88)	0.245				
**Hyperlipidemia**						
No	Ref.					
Yes	2.10 (0.54-8.19)	0.285				
**Thiazide**						
No	Ref.					
Yes	0.32 (0.01-8.78)	0.504 [a]				
**Benzbromarone**						
No	Ref.		Ref.		Ref.	
Yes	36.98 (4.32-316.64)	0.001	5.02 (0.60-42.11)	0.137	6.29 (0.76-52.27)	0.089

[a] Firth logistic regression
